
JOURNAL INFORMATION
==============================
NLM Title Abbreviation: ScientificWorldJournal
Journal ID: ScientificWorldJournal
NLM Journal ID: 101131163
Journal ID: TSWJ

The Scientific World Journal

ISSN: 2356-6140
EISSN: 1537-744X
Publisher: Wiley

ARTICLE INFORMATION
==============================
PMCID: PMC5974768
PMID: 12847302
DOI: 10.1100/tsw.2003.38
Article ID: 548520
Article version: 1
Subjects: Directions in Science

Ras Signalling Pathway: A Gateway for HSV-1 Infection

Farassati Faris *
Lee Patrick W.K.
University of Calgary, Department of Microbiology and Infectious Diseases, 3330-Hopsital Dr. N.W., Calgary, AB, Canada
*Corresponding author.
Publication date: 2003
Electronic publication date: 2003 Jun 18
Volume: 3
First page: 533
Last page: 535
Copyright: Copyright © 2003 Faris Farassati and Patrick W.K. Lee.
Copyright year: 2003
License: This is an open access article distributed under the Creative Commons Attribution License, which permits unrestricted use, distribution, and reproduction in any medium, provided the original work is properly cited.
License URL: https://creativecommons.org/licenses/by/3.0/

==============================
Directions in Science
*TheScientificWorldJOURNAL* (2003) 3, 533-535
ISSN 1537-744X; DOI 10.1100/tsw.2003.38

### Ras Signalling Pathway: A Gateway for HSV-1 Infection

Faris Farassati* and Patrick W.K. Lee
*University of Calgary, Department of Microbiology and Infectious Diseases, 3330-Hopsital Dr. N.W., Calgary, AB, Canada T2N4N1*

*Received May 29, 2002; Revised May 19, 2003; Accepted May 23, 2003; Published June 18, 2003*

---

The key role of Ras in diverse cellular functions qualifies it as a central divergence point for many signal transduction pathways, the most extensively studied of which include the ERK (extracellular signal-regulated kinase), JNK (c-Jun N-terminal kinase), and p38-kinase pathways[1,2]. Stimulation of these pathways results in the activation of different nuclear transcription factors (including SRF, ELK-1, JUN, NFκB, and ATF-2), which in turn leads to a variety of biological functions regulating cell growth, differentiation, apoptosis, migration, and survival[3,4].

In this study, a new role for Ras signaling pathway as key determinant of host cell permissiveness to viral infection was investigated. Herpes simplex virus-1 (HSV-1) was chosen because of its importance as a human pathogen and its potential use in cancer therapy[5,6,7,8,9].

The authors initially compared the level of viral infection in untransformed NIH-3T3 cells with that in cells transformed with oncogenes that activate the Ras pathway (e.g., v-*erbB*, *sos,* and *ras)*. Only transformed cells demonstrate significant morphological changes as a result of infection (such as cell rounding and clumping), increased viral protein synthesis, and a high titer of viral progeny. Interestingly, this was specific to the cells with an overactivated Ras-pathway and was not a general feature of the transformed phenotype, as cells transformed with c-*myc* did not have an increase in viral protein synthesis. The demonstration that inhibitors of Ras (farnesyl transferase inhibitors or FTIs)[10] and a blocker of MEK1/2 (a downstream molecule in ERK pathway) named PD98059[11], prevented this increase in HSV-1 infection further proved that an activated Ras pathway is important for infection.

As Ras is a key regulator of several signalling pathways, mutant versions of Ras that preferentially activate only one of several known pathways downstream of Ras (RAF/ERK, PI3K, or RAL/GDS) were used to find out which pathway was involved in permissiveness to infection. The authors showed that the RAF/ERK pathway was the only one whose activation significantly increased susceptibility to HSV-1.

To further elucidate the role(s) of the Ras pathway in HSV-1 infection, it was important to identify the step at which HSV-1 infection is blocked in the nonpermissive NIH-3T3 cells. Since virus binding and internalization are comparable between permissive and nonpermissive cells, transcription of viral genes were considered to be a logical target for investigation. Herpes genes are transcribed in a sequential manner with α genes being the first to be expressed and required for the synthesis of subsequent polypeptide groups β and γ [5]. Accordingly, the relative amounts of HSV-1 transcripts (including the α gene α27, β genes $U_L29$ and $U_L30$, γ1 gene $\gamma_1 34.5$, and γ2 gene $U_L44$) generated in NIH-3T3 cells and their Ras-transformed version (named H-ras cells) were compared after amplification of these transcripts by quantitative RT-PCR. The results show

*Corresponding author. Current address: Massachusetts General Hospital-East, Harvard Medical School, 149-13th Street, Charlestown, MA, 02129
©2003 with author.

that while the immediate early $\alpha$ transcript $\alpha$27 accumulated to comparable levels in the two cell lines, the $\beta$ and $\gamma$ transcripts were present at significantly higher levels in the H-ras cells than in the untransformed NIH-3T3 cells. Since transcription of these later ($\beta$ and $\gamma$) genes requires early $\alpha$ gene products, the drastic reduction in $\beta$ and $\gamma$ gene expression in NIH-3T3 cells was likely due to the inability of the $\alpha$ transcripts to be efficiently translated. Indeed, Western blot analysis revealed that the $\alpha$27 gene product ICP27 was present at a much lower level in NIH-3T3 cells than H-ras cells, despite the presence of comparable amounts of $\alpha$27 transcripts in the two cell lines. Furthermore, accumulation of ICP8 (product of $U_L$29) and gC (product of $U_L$44) was observed in H-ras but not NIH-3T3 cells. It thus appears that the $\alpha$ transcripts were not efficiently translated in NIH-3T3 cells, which in turn led to the lack of progression of downstream events, resulting in abortive HSV-1 infection in these cells.

The authors then proceeded to investigate the mechanism by which the translation of $\alpha$ genes was inhibited in NIH-3T3 cells and not in H-ras cells. A well-known antiviral strategy of cells is the phosphorylation/activation of the double-stranded RNA-activated protein kinase (PKR) by viral transcripts, which leads to phosphorylation of the translation initiation factor eIF2$\alpha$ and therefore blocks its function in initiation of translation of viral transcripts[12,13]. In order to avoid this, some viruses have developed mechanisms to block PKR function. $\gamma_1$34.5 is an example of such “viral anti-PKR activity” which induces dephosphorylation of eIF2$\alpha$ and therefore antagonizes PKR function. In this study, *in vitro* kinase assays were used to demonstrate that PKR phosphorylation is more pronounced in untransformed cells as compared with H-ras cells. This was followed by western blotting to show that phosphorylation of eIF2$\alpha$ is also more pronounced in untransformed NIH-3T3 cells.

A mutant version of HSV-1, G207, is currently being tested in clinical trials as an anticancer therapeutic. G207 is a derivative of another mutant called R3616, which lacks both copies of $\gamma_1$34.5 gene and can be considered as an attenuated virus. R3616 was found to infect *ras*-transformed, but not untransformed, cells suggesting that the Ras signalling pathway can compensate for the loss of the virus’ anti-PKR mechanism. Confirming the role of PKR in this regard, mouse embryonic fibroblasts (MEFs) that were deleted for PKR gene could be infected by the mutant virus while parental MEFs remained unpermissive. As expected, the use of farnesyl transferase inhibitors caused a restoration in PKR phosphorylation, thereby promoting the resistance of H-ras cells to HSV infection.

HSV-1 therefore exploits the attenuating effect of the Ras pathway on the PKR system. Once PKR system is down regulated by overactivity of the Ras signalling pathway, the stage is set for infection of cells by oncolytic viruses. This study not only provides knowledge on the mechanism behind host-cell permissiveness to HSV-1, but also provides a news basis for application of Ras blocking agents for the treatment of HSV infections.

### REFERENCES

1. Paduch, M., Jelen, F., and Otlewski, J. (2001) Structure of small G proteins and their regulators. *Acta Biochim. Pol.* **48(4)**, 829–850.
2. Shields, J.M., Pruitt, K., McFall, A., Shaub, A., and Der, C.J. (2000) Understanding Ras: 'it ain't over 'til it's over'. *Trends Cell Biol.* **10(4)**, 147–154.
3. Hazzalin, C.A. and Mahadevan, L.C. (2002) MAPK-regulated transcription: a continuously variable gene switch? *Nat. Rev. Mol. Cell Biol.* **3,** 30–40.
4. Olson, M.F. and Marais, R. (2000) Ras protein signalling. *Semin. Immunol.* **12,** 63–73.
5. Whitley, R.J. and Roizman, B. (2001) Herpes simplex virus infections. *Lancet* **357(9267),** 1513–1518.
6. Andreansky, S.S., Soroceanu, L., Flotte, E.R., Chou, J., Markert, J.M., Gillespie, G.Y., Roizman, B., and Whitley, R.J. (1997) Evaluation of genetically engineered herpes simplex viruses as oncolytic agents for human malignant brain tumors. *Cancer Res.* **57,** 1502–1509.

7. Andreansky, S.S., He, B., Gillespie, G.Y., Soroceanu, L., Markert, J., Chou, J., Roizman, B., and Whitley, R.J. (1996) The application of genetically engineered herpes simplex viruses to the treatment of experimental brain tumors. *Proc. Natl. Acad. Sci. U. S. A.* **93**, 11313–11318.
8. Yazaki, T., Manz, H.J., Rabkin, S.D., and Martuza, R.L. (1995) Treatment of human malignant meningiomas by G207, a replication-competent multimutated herpes simplex virus 1. *Cancer Res.* **55**, 4752–4756.
9. Markert, J.M., Medlock, M.D., Rabkin, S.D., Gillespie, G.Y., Todo, T., Hunter, W.D., Palmer, C.A., Feigenbaum, F. Tornatore, C., Tufaro, F., and Martuza, R.L. (2000) Conditionally replicating herpes simplex virus mutant, G207 for the treatment of malignant glioma: results of a phase I trial. *Gene Ther.* **7(10),** 867–874.
10. Prendergast, G.C. and Rane, N. (2001) Farnesyltransferase inhibitors: mechanism and applications. *Expert Opin. Invest. Drugs* **10(12),** 2105–2116.
11. Dudley, D.T., Pang, L., Decker, S.J., Bridges, A.J., and Saltiel, A.R. (1995) A synthetic inhibitor of the mitogen-activated protein kinase cascade. *Proc. Natl. Acad. Sci. U. S. A.* **92**, 7686–7689.
12. Williams, B.R. (1999) PKR a sentinel kinase for cellular stress. *Oncogene* **18,** 6112–6120.
13. Clemens, M.J. and Elia, A. (1997) The double-stranded RNA-dependent protein kinase PKR: structure and function. *J. Interferon Cytokine Res.* **17**, 503–524.

**This article should be referenced as follows**:

Farassati, F. and Lee, P.W.K. (2003) Ras signalling pathway: a gateway for HSV-1 infection. *TheScientificWorldJOURNAL* **3**, 533–535.
